# DMAG, a novel countermeasure for the treatment of thrombocytopenia

**DOI:** 10.1186/s10020-021-00404-1

**Published:** 2021-11-27

**Authors:** Jing Lin, Jing Zeng, Sha Liu, Xin Shen, Nan Jiang, Yue-Song Wu, Hong Li, Long Wang, Jian-Ming Wu

**Affiliations:** 1 School of Pharmacy, Southwest Medical University, Luzhou, 646000 Sichuan China; 2 School of Pharmacy, Chengdu University of Traditional Chinese Medicine, Chengdu, 611137 Sichuan China; 3 Institute of Cardiovascular Research, The Key Laboratory of Medical Electrophysiology, Ministry of Education of China, Medical Key Laboratory for Drug Discovery and Druggability Evaluation of Sichuan Province, Luzhou Key Laboratory of Activity Screening and Druggability Evaluation for Chinese Materia Medica, Luzhou, 646000 China

**Keywords:** DMAG, Megakaryocytes, Platelets, Network pharmacology, Thrombocytopenia

## Abstract

**Background:**

Thrombocytopenia is one of the most common hematological disease that can be life-threatening caused by bleeding complications. However, the treatment options for thrombocytopenia remain limited.

**Methods:**

In this study, giemsa staining, phalloidin staining, immunofluorescence and flow cytometry were used to identify the effects of 3,3ʹ-di-*O*-methylellagic acid 4ʹ-glucoside (DMAG), a natural ellagic acid derived from *Sanguisorba officinalis* L. (SOL) on megakaryocyte differentiation in HEL cells. Then, thrombocytopenia mice model was constructed by X-ray irradiation to evaluate the therapeutic action of DMAG on thrombocytopenia. Furthermore, the effects of DMAG on platelet function were evaluated by tail bleeding time, platelet aggregation and platelet adhesion assays. Next, network pharmacology approaches were carried out to identify the targets of DMAG. Gene Ontology (GO) and Kyoto Encyclopedia of Genes and Genomes (KEGG) pathway enrichment analyses were performed to elucidate the underling mechanism of DMAG against thrombocytopenia. Finally, molecular docking simulation, molecular dynamics simulation and western blot analysis were used to explore the relationship between DAMG with its targets.

**Results:**

DMAG significantly promoted megakaryocyte differentiation of HEL cells. DMAG administration accelerated platelet recovery and megakaryopoiesis, shortened tail bleeding time, strengthened platelet aggregation and adhesion in thrombocytopenia mice. Network pharmacology revealed that ITGA2B, ITGB3, VWF, PLEK, TLR2, BCL2, BCL2L1 and TNF were the core targets of DMAG. GO and KEGG pathway enrichment analyses suggested that the core targets of DMAG were enriched in PI3K–Akt signaling pathway, hematopoietic cell lineage, ECM-receptor interaction and platelet activation. Molecular docking simulation and molecular dynamics simulation further indicated that ITGA2B, ITGB3, PLEK and TLR2 displayed strong binding ability with DMAG. Finally, western blot analysis evidenced that DMAG up-regulated the expression of ITGA2B, ITGB3, VWF, p-Akt and PLEK.

**Conclusion:**

DMAG plays a critical role in promoting megakaryocytes differentiation and platelets production and might be a promising medicine for the treatment of thrombocytopenia.

**Graphical Abstract:**

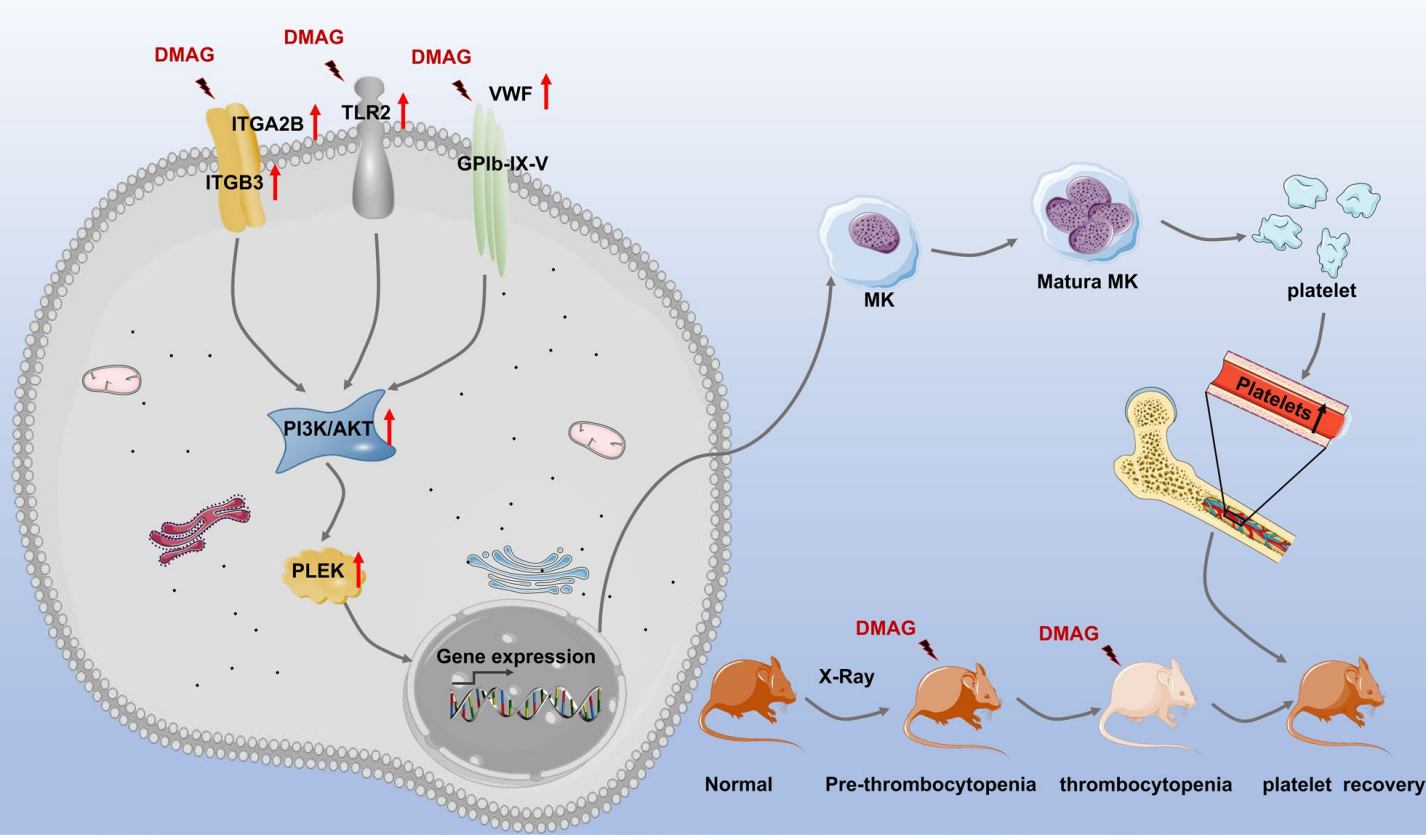

**Supplementary Information:**

The online version contains supplementary material available at 10.1186/s10020-021-00404-1.

## Introduction

Platelets, a major type of blood cells, play a critical role in hemostasis, inflammation, thrombosis and immunity (Eto and Kunishima 2016). These small anucleated cells are the final products of megakaryocytes differentiation and maturation. Megakaryocytes (MK) derive from hematopoietic stem cells (HSCs) and undergo a continuous and complex maturation program (Nishikii et al. 2015). Megakaryocytes increase their size and become polyploid through repeating cycles of DNA replication without cell division, a process called endomitosis (Mattia et al. 2002). In this process, the internal membrane systems of megakaryocytes gradually maturate (Eckly et al. 2014), F-actin gradually aggregates (Poulter and Thomas 2015), and the specific surface antigens for megakaryocyte differentiation are gradually expressed, such as CD41, CD61, CD42b and Von Willebrand factor (VWF) (Tomer 2004; Dumon et al. 2012). At the end, megakaryocytes extend the voluminous protrusions into the lumen of sinusoids, and under the shearing force of flowing blood, the protrusions are cut off and platelets shed into the blood (Patel et al. 2005). Various cytokines, chemokines, signaling pathways and transcription factors regulate MK differentiation through transcription and microenvironmental mechanisms at multiple levels. Thrombopoietin (TPO) is the most important mediators of megakaryopoiesis. It binds its receptor, c-MPL, activating a number of downstream signaling pathways, including JAK2/STATs, MAPK/ERK, and PI3K/Akt signaling pathway (Bianchi et al. 2016; Eto and Kunishima 2016). Since megakaryocytes are located in bone marrow (BM), the BM microenvironment plays a vital role in both megakaryocyte differentiation and maturation, and platelet production (Leiva et al. 2018). For instance, the BM extracellular matrix (ECM) is an acellular component that provides physical support for hematopoiesis. The ECM matrix proteins, such as VWF, fibrinogen, fibronectin, collagen type IV and laminin bind to corresponding receptors of megakaryocyte, and regulate proplatelet reorganization and platelet formation (Guo et al. 2015).

Any abnormity in the process of megakaryocyte differentiation and maturation, as well as platelet release can lead to platelets disorders (Krishnegowda and Rajashekaraiah 2015). Particularly, thrombocytopenia, an important clinical problem that may cause by either impaired megakaryocyte maturation or insufficient platelets production (Greenberg 2017). The severe thrombocytopenia can cause intra-cerebral and intra-abdominal bleeding, which can be fatal. Currently, platelet transfusion is the most direct and effective method for treating thrombocytopenia (Stroncek and Rebulla 2007). However, due to the platelets shortage and the common occurrence of allergic transfusion reactions (ATRs), the clinical application of platelet transfusion is largely limited (Wang et al. 2017). Promoting the body’s own platelets production has become the effective strategy for clinical intervention of thrombocytopenia. The most widely used drugs for increasing the level of patients’ platelets is thrombopoietin receptor agonists (TPO-RA), such as Eltrombopag and Romiplostim. But the drugs are not only expensive but also can increase the risk of thromboembolic complications (Garnock-Jones 2011; Yuan et al. 2019). Therefore, it is urgent and necessary to discovery novel therapeutic drugs with high efficacy and safety for the treatment of thrombocytopenia.

*Sanguisorba officinalis* L. (SOL) has been used as herbal medicine for treating hemorrhage syndrome for a long time in Asia and Europe (Gawron-Gzella et al. 2016; Bai et al. 2019). In recent decades, SOL was used to treat myelosuppression induced by chemotherapy or radiotherapy in clinic (Ma et al. 2015). Our previous study demonstrated that the ethanol extract of SOL exhibited remarkable therapeutical effect against leukopenia in mice (Wang et al. 2020). We also found that two ellagic acids, 3,3ʹ,4-tri-*O*-methylellagic acid-4ʹ-*O*-β-d-xyloside and 3,3ʹ,4-tri-*O*-methylellagic acid isolated from SOL significantly stimulated hematopoietic progenitor cell proliferation and megakaryocyte differentiation  (Gao et al. 2014). However, the active compounds of SOL in promoting hematopoietic recovery and their underling molecular mechanism remain largely unknown. In the present study, we aim to investigate the effects of DMAG on megakaryocyte differentiation in vitro, and evaluate the therapeutic action on thrombocytopenia in vivo. We further seek to elucidate the molecular mechanism of DMAG in the treatment of thrombocytopenia using network pharmacology method and experimental verification.

## Materials and methods

### Chemicals

DMAG, 3,3ʹ-di-*O*-methylellagic acid 4ʹ-glucoside (CAS:51803-68-0, purity ≥ 98%) was obtained from Chengdu Push Bio-technology Co., Ltd (Chengdu, China).

### Cell culture

HEL cells were purchased from American Type Culture Collection (Rockville, MA, USA). The cells were cultured in RPMI 1640 medium, supplemented with 10% fetal bovine serum (FBS) and 1% penicillin–streptomycin at 37 °C in a humidified atmosphere with 5% CO_2_.

### Giemsa staining

4.0 × 10^4^ HEL cells were seeded in 6-well plates and were treated with DMAG (10, 20 and 40 μM) for 6 days. The cells were harvested and washed with PBS for twice. Then cells were fixed with fixing solution (methanol:glacial acetic acid = 3:1 (v/v)), and stained with Giemsa solution (Solarbio, Beijing, China) for 5 min. The stained cells were finally photographed under electron microscope (10×).

### Phalloidin staining

After treatment for 6 days, cells were harvested for F-actin staining by phalloidin staining (Solarbio, Beijing, China) according to the manufacturer’s instructions. In brief, the cells were fixed with 4% paraformaldehyde for 15 min and permeabilized with 0.05% Triton X-100 for 10 min at the room temperature. After that, cells were washed twice with PBS and TRITC-conjugated Phalloidin (1:200) was performed for 30 min in dark at room temperature, then DAPI was added to counterstain the nucleus for 5 min. Finally, the representative images were captured using the inverted fluorescence microscope (Nikon Ts2R/FL, Japan).

### Immunofluorescence assay

After treating with DMAG (10, 20 and 40 μM) for 6 days, HEL cells were harvested and washed with PBS for twice. Then cells were fixed with 4% paraformaldehyde for 15 min and permeabilized with 0.05% Triton X-100 for 10 min at room temperature. Cells were washed twice with PBS and incubated with anti-VWF antibody (Proteintech, IL, USA, 11778-1-AP, 1:200) overnight at 4 °C. After a thorough wash with PBS, FITC-conjugated secondary antibody (ZSGB-BIO, Beijing, China, ZF-0311, 1:100) was added to against with primary antibody, and DAPI was added to counterstain the nucleus. The representative images were captured using the inverted fluorescence microscope (Nikon Ts2R/FL, Japan).

### Flow cytometry analysis the co-expression of CD41/CD42b and CD41/CD61

After treating with DMAG (10, 20 and 40 μM) for 6 days, HEL cells were harvested and washed with PBS for twice, then labeled with human anti-CD41-FITC (4A Biotech, Beijing, China, FHF0411-100), human anti-CD42b-PE (BD Pharmingen, CA, USA, 555473) and human anti-CD61-PE (BioLegend, CA, USA, 336406) antibody on ice in the dark for 30 min. The samples were resuspended in 400 μL PBS for analysis by flow cytometry (BD Biosciences, San Jose, CA, USA).

### Megakaryocytes ploidy assay

HEL cells were treated with DMAG (10, 20 and 40 μM) for 6 days and then harvested for DNA ploidy analysis using CycleTEST™ PLUS DNA Reagent Kit (Cycletest Plus DNA Reagent, BD) according to the manufacturer’s instructions. Briefly, HEL cells were harvested and resuspended in buffer solution. After incubation with trypsin buffer for 10 min, cells were treated with trypsin inhibitor and RNase buffer for 10 min at room temperature. Then cells were incubated with PI stain solution for 10 min in the dark and flow cytometry was performed by the BD FACSCanto II flow cytometer (BD Biosciences, San Jose, CA, USA). Despite flow cytometry is widely used to analyze the cell cycle and DNA ploidy, high content screening (HCS) fills the gap of flow cytometry in modern high-resolution imaging technology. It builds a bridge between image acquisition and image analysis, and focuses on the construction of DNA content and cell cycle parameters (Furia et al. 2013, 2014; Schorpp et al. 2016). Therefore, we used the cell cycle analysis module of HCS to detect the megakaryocytes ploidy again. In brief, HEL cells were collected and washed twice with PBS, then cells were transferred to a 96-well plate at a density of 2 × 10^5^ cells/well. DAPI (100 nM, Solarbio, Beijing, China) was added and incubated at room temperature in the dark for 10 min. Lastly, the sample was detected by the ImageXpress Micro4 (Molecular Devices, San Jose, CA, USA).

### Establishment the thrombocytopenia mice model and treatment with DMAG

Specifc-pathogen-free Kunming mice (KM), 8 to 10-week-old, were purchased from Da-shuo Bio-technology Limited (Chengdu, Sichuan, China). The mice were maintained under standard condition (22 ± 2 °C, 55 ± 5% humidity and 12 h light/dark cycle). All experimental procedures were approved by the laboratory animal ethics committee of the Southwest Medical University (Luzhou, China). Except for the control group, the other mice were irradiated by X-ray (4 Gy) to establish thrombocytopenia mice model. According to the level of peripheral blood, the mice were randomly divided into four groups (6 male mice and 6 female mice in each group): control group, model group, TPO positive group, DMAG group. The mice in control group and model group were intraperitoneally administered with normal saline per day. The mice in TPO positive group and DMAG group were intraperitoneally administered with TPO (3000 U/kg) or DMAG (5 mg/kg) per day and lasted for 14 days.

### Measurement of hematologic parameters

On day 0, 3, 7, 10, and 14, the blood was collected from eyes’ venous plexus for hematologic parameters analysis by Hematology Analyzer (SYSMEX XT-1800Iv; Kobe, Japan).

### Histology and immunohistochemistry

On day 10, the femurs were collected and fixed in 10% formaldehyde for 24 h. After decalcification for a month, the femurs were embedded in paraffin and cut into 5 µm thick sections. Then the samples were stained with hematoxylin and eosin (H&E) or anti-VWF antibody (Proteintech, IL, USA, 11778-1-AP, 1:100). Images were captured using Olympus BX51microscope (Olympus Optical).

### Flow cytometry analysis for megakaryocyte differentiation in bone marrow

On day 10, BM cells were collected and counted by Hematology Analyzer to adjust the cell density at 100 × 10^4^ cells per sample. Mouse anti-CD41-FITC (Biolegend, CA, USA, 133904) and mouse anti-CD61-PE (Biolegend, CA, USA, 104308) were added into cells and incubated for 15 min in dark on ice. After that, the samples were resuspended in 1 mL PBS for analysis by the BD FACSCanto II flow cytometer (BD Biosciences, San Jose, CA, USA).

### Tail bleeding time

2 mm of the distal tail from non-anesthetized mice was cut and immediately immersed in normal saline at 37 ℃. The bleeding time was counted as described previously (Meinders et al. 2015).

### Platelet aggregation

Whole blood was collected from the inferior vena cava of anesthetized mice and anticoagulated with 3.8% sodium citrate. Then, platelet-rich plasma (PRP) and platelet-poor plasma (PPP) were obtained by centrifugation at 100*g* and 2000*g* for 10 min at 22 ℃, respectively. PRP was counted by Hematology Analyzer to adjust platelet density at 300 × 10^9^ per sample. After the stimulation of ADP (Helena Laboratories, USA), platelet aggregation was analyzed with a turbidimetric aggregation-monitoring device (Helena Laboratories, Beaumont, TX, United States) according to previously report (Li et al. 2020).

### Platelet adhesion

For the preparation of plates coated with collagen, 250 μL of 5 μg/mL collagen (Helena Laboratories, USA) was added to the bottom of each confocal dish and kept overnight at 4 ℃. After washing with PBS, each well was blocked with 1% BSA for 1 h at room temperature. PRP (2 × 10^7^ cell/mL) was then added to each well and incubated at 37 °C for 45 min. After unbound platelets were removed by PBS, wells were fixed with 4% paraformaldehyde for 15 min and permeabilized with 1% Triton X-100 for 10 min at room temperature. After that, the adhering platelets were stained with TRITC-conjugated phalloidin (1:200) (Suzuki-Inoue et al. 2001). The representative images were captured using the inverted fluorescence microscope (Nikon Ts2R/FL, Japan). The average coverage of adhering platelets was calculated by ImageJ software.

### Acquisition of candidate targets of DMAG against thrombocytopenia

PharmMapper (https://lilab.ecust.edu.cn/pharmmapper/index.php) and Swiss database (http://www.swisstargetprediction.ch/index.php) were used to identify targets of DMAG. The GeneCards database (https://www.genecards.org/) and DisGeNET database (http://www.disgenet.org/) were used to retrieve targets related to thrombocytopenia. The common targets of DMAG with thrombocytopenia were considered as potential targets of DAMG against thrombocytopenia. Venn diagram was drawn on Jvenn website (http://jvenn.toulouse.inra.fr/app/example.html) to obtain the overlapped targets of DMAG with thrombocytopenia. The component-target-disease network was constructed by cytoscape_v3.7.1 software.

### Construction of protein–protein interaction (PPI) network and identification of core targets of DMAG against thrombocytopenia

PPI network was constructed by STRING database (http://string-db.org) and visualized by Cytoscape_v3.7.1 software. The screening conditions of core targets of DMAG against thrombocytopenia were as follows: Degree was greater than or equal to twice the median, Betweenness Centrality (BC) and Closeness Centrality (CC) were greater than or equal to the median.

### GO and KEGG pathway analyses of core targets

Database for Annotation, Visualization and Integrated Discovery database (DAVID, https://david.ncifcrf.gov/) was used to obtain GO and KEGG pathway analyses. Visualization of GO and KEGG pathway analyses by using GraphPad Prism v9.1.0.221 software and OmicShare website (https://www.omicshare.com/tools/).

### Molecular docking simulation and molecular dynamics simulation

Molecular docking simulation was used to explore the binding ability between DMAG and its core targets. The crystal structures of core targets were obtained from the RCSB Protein Data Bank (https://bivi.co/visualisation/rcsb-protein-data-bank). Sybyl-X 2.0 software was used for structural modification of these structures, including residue modification and repair, hydrogenation and charging. The 3D structure of DMAG was constructed based on the PubChem database (https://pubchem.ncbi.nlm.nih.gov), its partial atomic charges were calculated by the Gasteiger Hückel method, energy minimizations were performed using the Tripos force field and the Powell conjugate gradient algorithm convergence criterion of 0.01 kcal/mol Å. After binding pocket was generated using the Protomol generation technique of SYBYL, the molecular docking between DMAG and core proteins were simulated by Sybyl-X 2.0 (Ragunathan et al. 2018). Molecular docking simulation was visualized utilizing Pymol and Ligplus software. Molecular dynamics simulation is a widely used tool to explore the dynamic binding of compounds to proteins. We used the DMAG-protein complex obtained by molecular docking to establish a molecular dynamics model using AMBER18 software. Then, the biological macromolecule system was optimized, and the conditions were set as follows: the DMAG-protein complex was dissolved in the TIP3P water model containing H_2_O, Na^+^ ions and Cl^+^ ions, and the temperature was heated to 300 K. After all optimizations are completed, a continuous simulation of 25 ns will be performed.

### Western blotting

HEL cells were collected after treated with DMAG (10, 20 and 40 μM) for 4 days. Total protein was extracted by RIPA lysis buffer (CST, MA, UAS) supplemented with protease inhibitors (Sigma, St Louis, MO). Protein was quantified with the Quick Start™ Bradford 1× Dye Protein Assay Reagent (Bio-Rad, CA, USA). An equal amount of protein (30 μg) was separated by sodium dodecyl sulfate polyacrylamide gel electrophoresis (SDS-PAGE) and transferred to a polyvinylidene fluoride (PVDF) membrane. After blocking with 5% skim milk powder in phosphate-buffered saline (PBS) for 60 min, the membrane was incubated with primary antibodies overnight at 4 °C followed by the HRP-bound secondary antibody for 60 min at 37 °C. The protein bands were visualized with ECL Western Blotting detection reagent (4A Biotech Co., Ltd., Beijing, China) and detected by the ChemiDoc MP Imaging System (Bio-Rad, Hercules, CA, USA). The proteins were quantified with ImageJ software. Primary antibodies were as follows: β-actin (CST, MA, USA, 3700S, 1:1000), ITGB3 (Proteintech, IL, USA, 18309-1-AP, 1:1000), ITGA2B (Proteintech, IL, USA, 24552-1-AP, 1:1000), PLEK (Proteintech, IL, USA, 12506-1-AP, 1:1000), VWF (Proteintech, IL, USA, 11778-1-AP, 1:500), P-Akt (CST, MA, USA, 4060S, 1:2000), Secondary antibodies were as follows: Mouse Anti-rabbit IgG (CST, MA, USA, 5127S, 1:2000). A β-actin antibody was used as a control.

## Result

### DMAG induces the differentiation of HEL cells

**Fig. 1:**
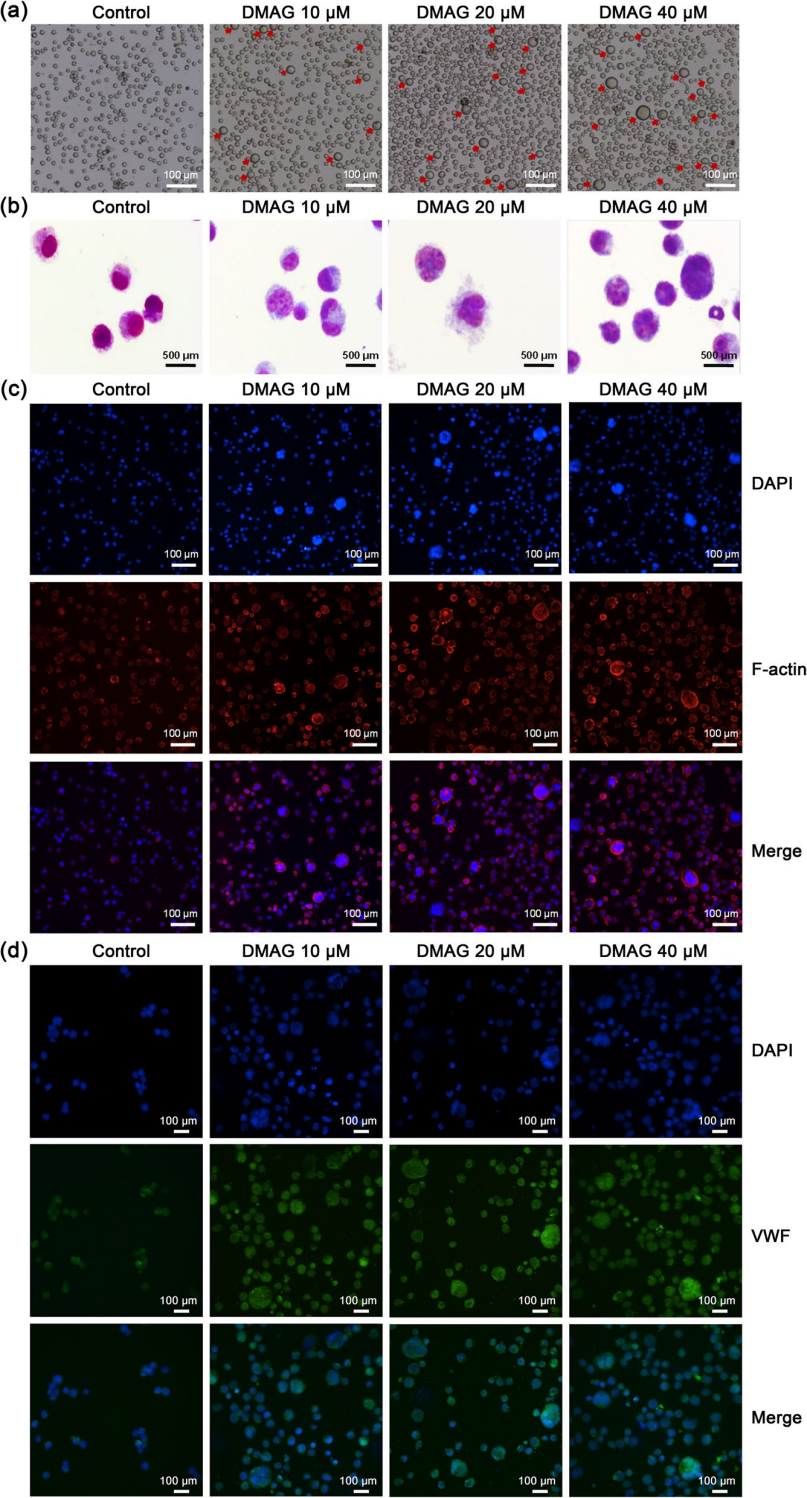
Morphological changes of HEL cells influenced by DMAG. **a** Morphological changes of HEL cells induced by DMAG (10, 20 and 40 μM) for 6 days. Scale bar: 100 μm. **b** Giemsa staining showed the multinucleation of HEL cells treated with DMAG (10, 20 and 40 μM) for 6 days. The dark blue represents cell nucleus. Scale bar: 500 μm. **c** The expression of F-actin of HEL cells is shown with phalloidin staining after the cells were treated with DMAG (10, 20 and 40 μM) for 6 days. Scale bar: 100 μm. Blue represents cell nucleus. Red indicates F-actin. **d** Immunofluorescence analysis of the expression of VWF after DMAG (10, 20 and 40 μM) intervention for 6 days in HEL cells. VWF was visualized by green fluorescence and cell nucleus was stained with DAPI (blue). Scale bar: 100 μm

**Fig. 2:**
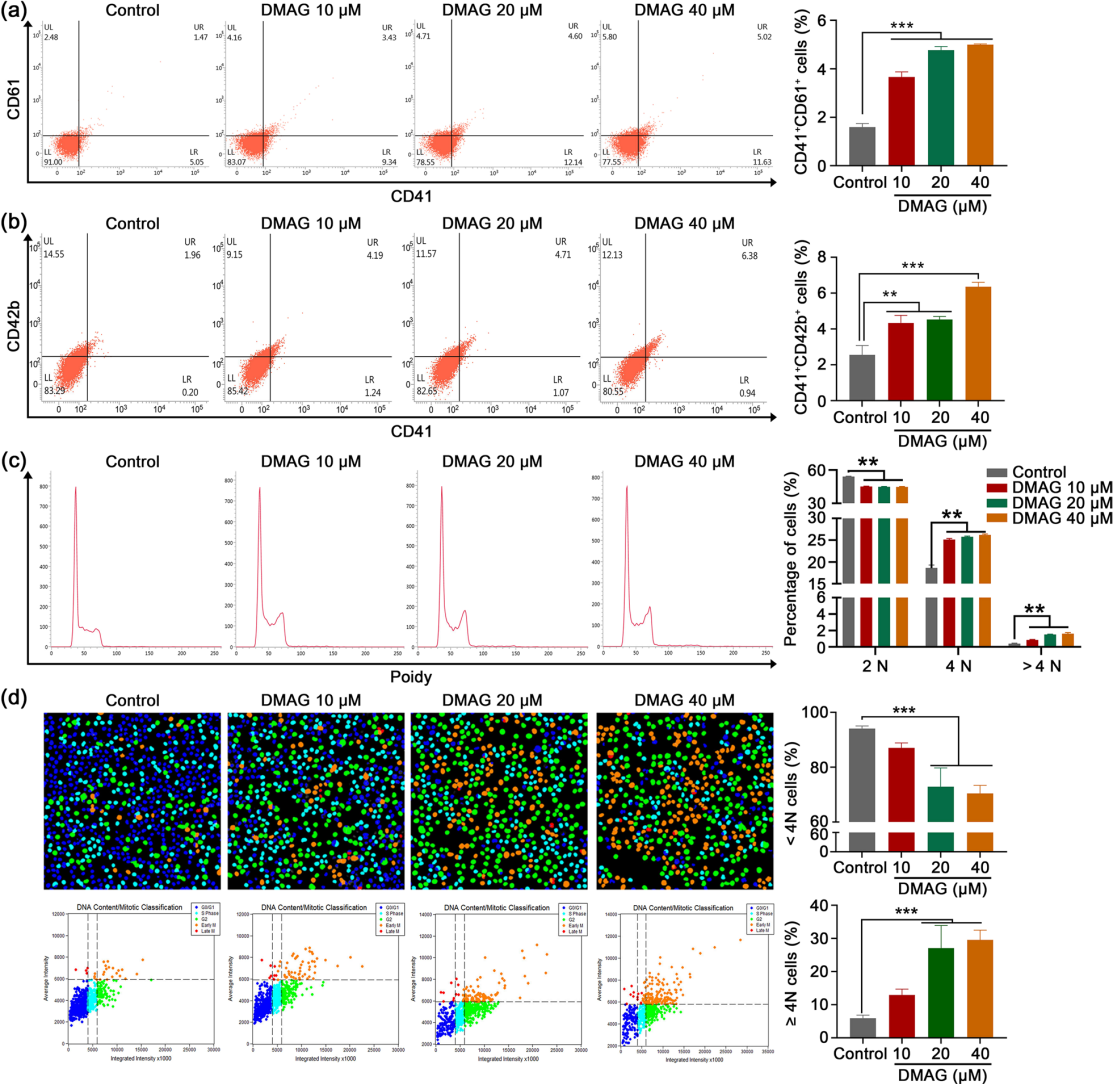
DMAG induces the differentiation of HEL cells. **a** The expression of CD41 and CD61 was detected by flow cytometry after the HEL cells were treated with DMAG (10, 20 and 40 μM) for 6 days. The histogram shows the percentage of CD41^+^CD61^+^ cells in each group. The data represent the mean ± SD of three independent experiments; ***p < 0.001, compared to the control group. **b** The expression of CD41 and CD42b was measured by flow cytometry after the HEL cells were treated with DMAG (10, 20 and 40 μM) for 6 days. The histogram shows the percentage of CD41^+^CD42b^+^ cells in each group. The data represent the mean ± SD of three independent experiments; **p < 0.01, ***p < 0.001, compared to the control group. **c** Flow cytometry analysis of DNA ploidy after the cells treated with DMAG (10, 20 and 40 μM) for 6 days. The histogram shows the percentage of DNA ploidy in each group. The data represent the mean ± SD of three independent experiments; **p < 0.01, ***p < 0.001, compared to the control group. **d** HCS analysis of DNA ploidy after the cells treated with or without DMAG (10, 20 and 40 μM) for 6 days. Cells were marked with blue, turquoise, green, orange and red. The different colors represent different phases of the cell cycle. Blues indicate G0/G1 (2 N) cells. Turquoises indicate S (2 N) cells. Greens represent G2 (≥ 4 N) cell. Oranges represent early M (≥ 4 N) cells. Reds represent late M (2 N) cells. The histogram shows the percentage of DNA ploidy in each group. The data represent the mean ± SD of three independent experiments; ***p < 0.001, compared to the control group

Given that ellagic acids derived from SOL displayed high activity in promoting megakaryocyte differentiation in our previous study, the effect of DMAG on the differentiation of HEL cells was assessed in the present study. We found another ellagic acids, DMAG, derived from SOL (Additional file 1: Fig. S1), that a large number of big cells appeared in DMAG (20 and 40 μM)-treated groups, while little in control group after 6 days of culture (Fig. 1a). Giemsa staining and phalloidin staining showed that cells treated with DMAG (20 and 40 μM) had larger size and more nucleus than that of control group (Fig. 1b, c). VWF is primarily synthesized in mature megakaryocytes and stored in platelet α-granules (Schick et al. 1997; Shi et al. 2003). Immunofluorescence showed that the expression of VWF was significantly enhanced after DMAG treatment (Fig. 1d). Then the expression of megakaryocytes-specific marker CD41 and CD61, and maturation marker CD42b was detected by flow cytometry. The results showed that the expression of CD41, CD61 and CD42b were all significantly increased in DMAG-treated groups in a concentration-dependent manner compared with control group (Fig. 2a, b). Moreover, ploidy assay revealed that DMAG treatment increased the DNA ploidy in a concentration-dependent manner (Fig. 2c). Correspondingly, high-content assay showed the similar results with ploidy assay detected by flow cytometry (Fig. 2d). Taken together, above data suggest that DMAG has the ability to induce megakaryocyte differentiation of HEL cells.

### DMAG possesses great therapeutic effects on mice with thrombocytopenia

**Fig. 3:**
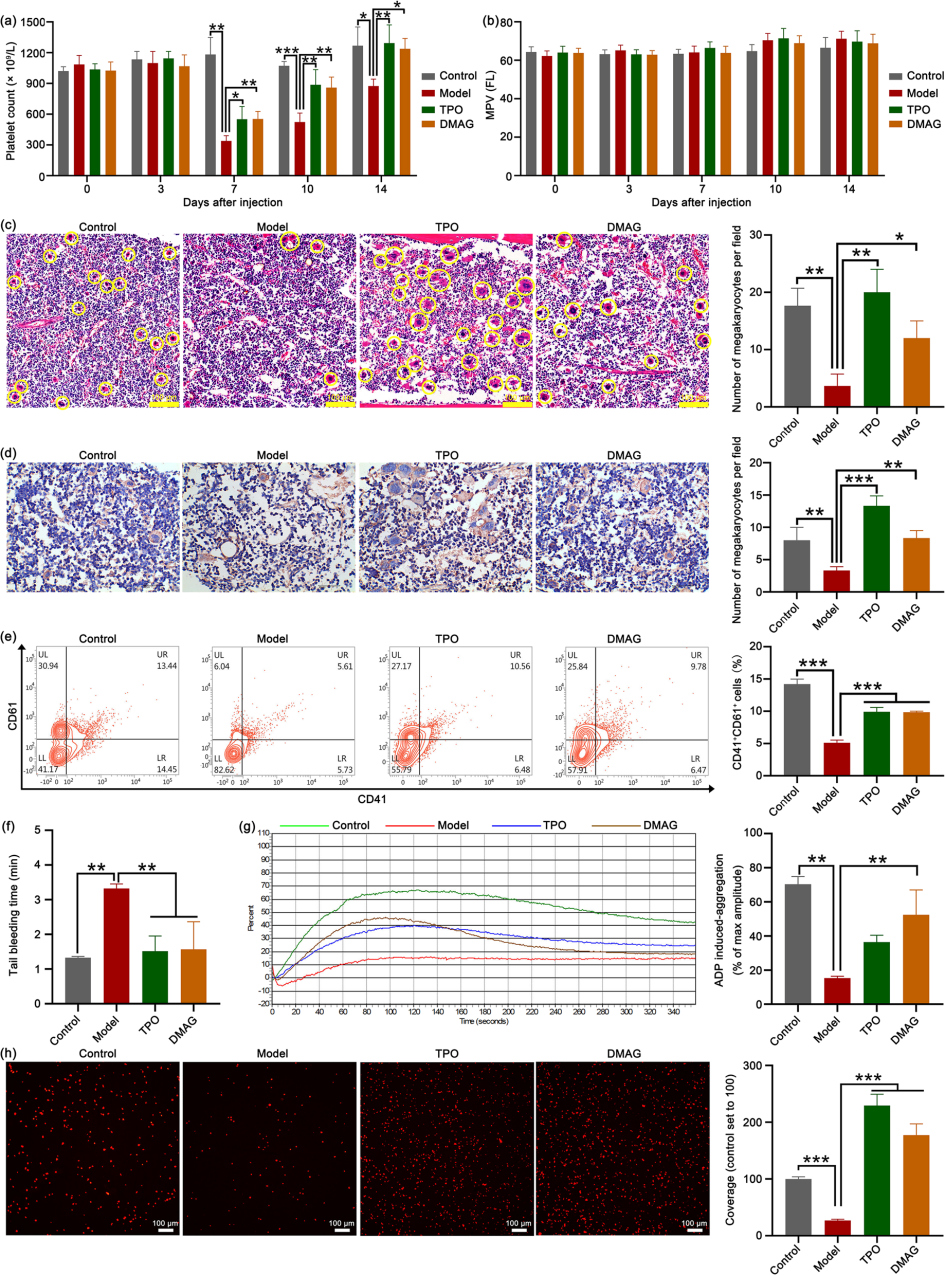
DMAG administration enhances recovery of platelet level and function in mice with thrombocytopenia. **a** The KM mice were irradiated by X-ray (4 Gy) and then treated with normal saline, TPO (3000 U/kg), or DMAG (5 mg/kg) for 14 days, respectively. The number of platelets were counted on day 0, 3, 7, 10, and 14. Each group contains 12 mice (6 male mice and 6 female mice). The data represent the mean ± SD of three independent experiments; *p < 0.05, **p < 0.01, ***p < 0.001, compared to the corresponding model group. **b** MPV was measured on day 0, 3, 7, 10, and 14 in each group. **c** Representative images of H&E staining of BM from normal, model, TPO and DMAG groups. The yellow circles indicate megakaryocytes. Scale bar: 100 μm. The histogram indicates the number of megakaryocytes in each group. The data represent the mean ± SD of three independent experiments; *p < 0.05, **p < 0.01, compared to the model group. **d** Immunohistochemical analysis of the expression of VWF in BM cells of each group. Blue represents cell nucleus. Brownness represents the expression of VWF. The histogram indicates the number of megakaryocytes in each group. The data represent the mean ± SD of three independent experiments; **p < 0.01, ***p < 0.001, compared to the model group. **e** The expression of CD41 and CD61 was detected by flow cytometry in BM cells of each group. The histogram indicates the percentage of CD41^+^CD61^+^ cells in each group. The data represent the mean ± SD of three independent experiments; ***p < 0.001, compared to the model group. **f** Tail bleeding time was measured after the distal tail was cut in each group. The data represent the mean ± SD of three independent experiments; **p < 0.01, compared to the model group. **g** Under the stimulation of ADP, platelet aggregation was measured by a turbidimetric aggregation-monitoring device. The histogram shows the maximum aggregation amplitude of platelets in each group. The data represent the mean ± SD of three independent experiments; **p < 0.01, compared to the model group. **h** Micrographs of collagen-coated slides with the same number of platelets perfused. The red represents platelets. The histogram shows the average coverage of red fluorescence on the whole surface by ImageJ software. The data represent the mean ± SD of three independent experiments; ***p < 0.001, compared to the model group

In order to evaluate the therapeutical effect of DMAG on thrombocytopenia, the thrombocytopenia mice model was constructed by 4 Gy X-ray total body irradiation. The results showed that the platelet count in all irradiation groups reached the lowest point on day 7 (Fig. 3a). However, the platelet count in DMAG-treated group and TPO group was significant higher compared with the model group (Fig. 3a), indicating that DMAG administration decelerated the rate of descent of platelet after irradiation damage. The platelet counts gradually recover after reaching the lowest point and the platelet count in DMAG-treated group and TPO group was higher than that of the model group (Fig. 3a), indicating that DMAG administration enhanced platelet recovery when the mice encountered irradiation. The mean platelet volume (MPV) was also detected and showed no difference between each group (Fig. 3b), indicating that DMAG had no effect on MPV. H&E staining results showed that DMAG treatment significantly increased the number of BM megakaryocytes (Fig. 3c). Immunohistochemistry data demonstrated that the number of VWF-positive megakaryocytes increased after DMAG treatment (Fig. 3d). Moreover, the expression of CD41 and CD61 of BM cells was measured by Flow cytometry. The results showed that DMAG administration obviously promoted the expression of CD41 and CD61, which demonstrated that DMAG could facilitate BM megakaryocyte differentiation. These results suggest that DMAG administration accelerates platelet recovery and megakaryocyte differentiation in mice with thrombocytopenia.

In order to explore whether the platelets produced by DMAG treatment were functional, we further evaluated platelet function in vivo and in vitro, respectively. Mouse tail bleeding model was used to measure the hemostatic function of platelets. The results showed that the tail bleeding time of mice in DMAG-treated group and TPO group was significantly shorter than that in the model group (Fig. 3f), suggesting that DMAG administration enhanced the hemostatic ability in thrombocytopenic mice. Furthermore, the platelet aggregation induced by ADP and platelet adhesive on solidified collagen were evaluated. The results showed that platelet aggregation induced by ADP was significantly strengthened in DMAG-treated when compared with the model group (Fig. 3g). Similarly, after DMAG or TPO treatment, platelet adhesion both improved (Fig. 3h). The above results suggest that DMAG administration is able to restore platelet function in thrombocytopenic mice. Taken together, these data demonstrate that DMAG exhibits good therapeutic effects on mice with thrombocytopenia.

### Identification of core targets of DMAG against thrombocytopenia

**Fig. 4:**
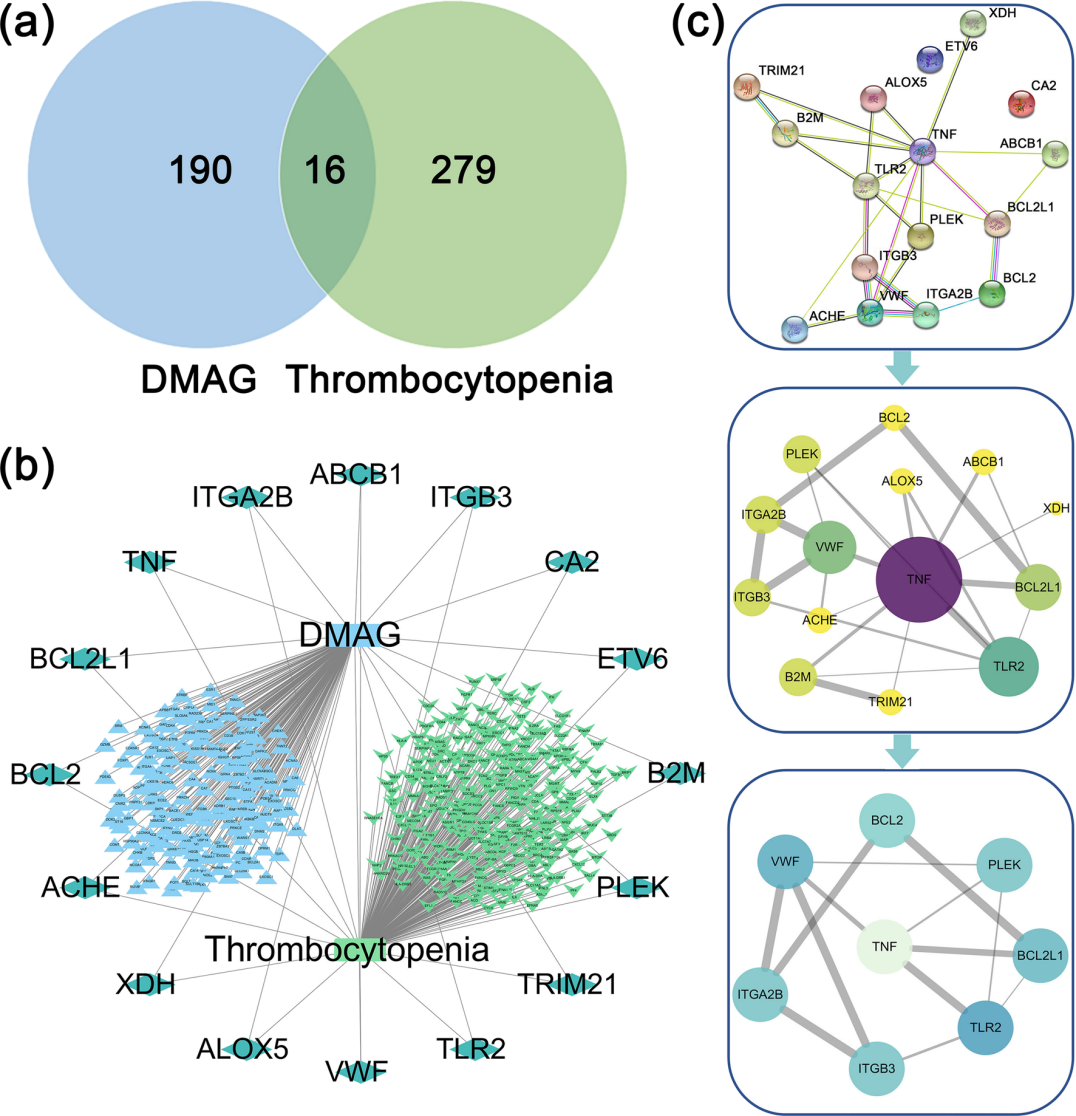
Prediction of the targets of DMAG against thrombocytopenia by network pharmacology. **a** Venn diagram shows the common targets of DMAG and thrombocytopenia. Blue and green regions represent DMAG associated and thrombocytopenia associated targets, respectively. The intersecting part represents the common targets between DMAG and thrombocytopenia. **b** The DMAG-targets-thrombocytopenia network that was constructed by cytoscape_v3.7.1 software. **c** PPI network for identifying core targets of DMAG against thrombocytopenia through the screening conditions of Degree > 2, BC > 0.0042735, CC > 0.43333333

The targets of DMAG against thrombocytopenia were predicted by network pharmacology. Through databases screening, a total of 206 targets were identified as targets of DMAG and 295 targets as targets related to thrombocytopenia. 16 common targets were considered as potential targets of DMAG for the treatment of thrombocytopenia (Fig. 4a, b). The core targets were screened out through two screenings: using Cytoscape_v3.7.1 software, first remove the two unrelated targets, and then set the screening conditions of Degree > 2, BC > 0.0042735, CC > 0.43333333. The top eight target proteins of ITGA2B, ITGB3, VWF, PLEK, TNF, TLR2, BCL2, BCL2L1 were regarded as core proteins (Fig. 4c).

### Enrichment analysis of the core targets of DMAG acting on thrombocytopenia

**Fig. 5:**
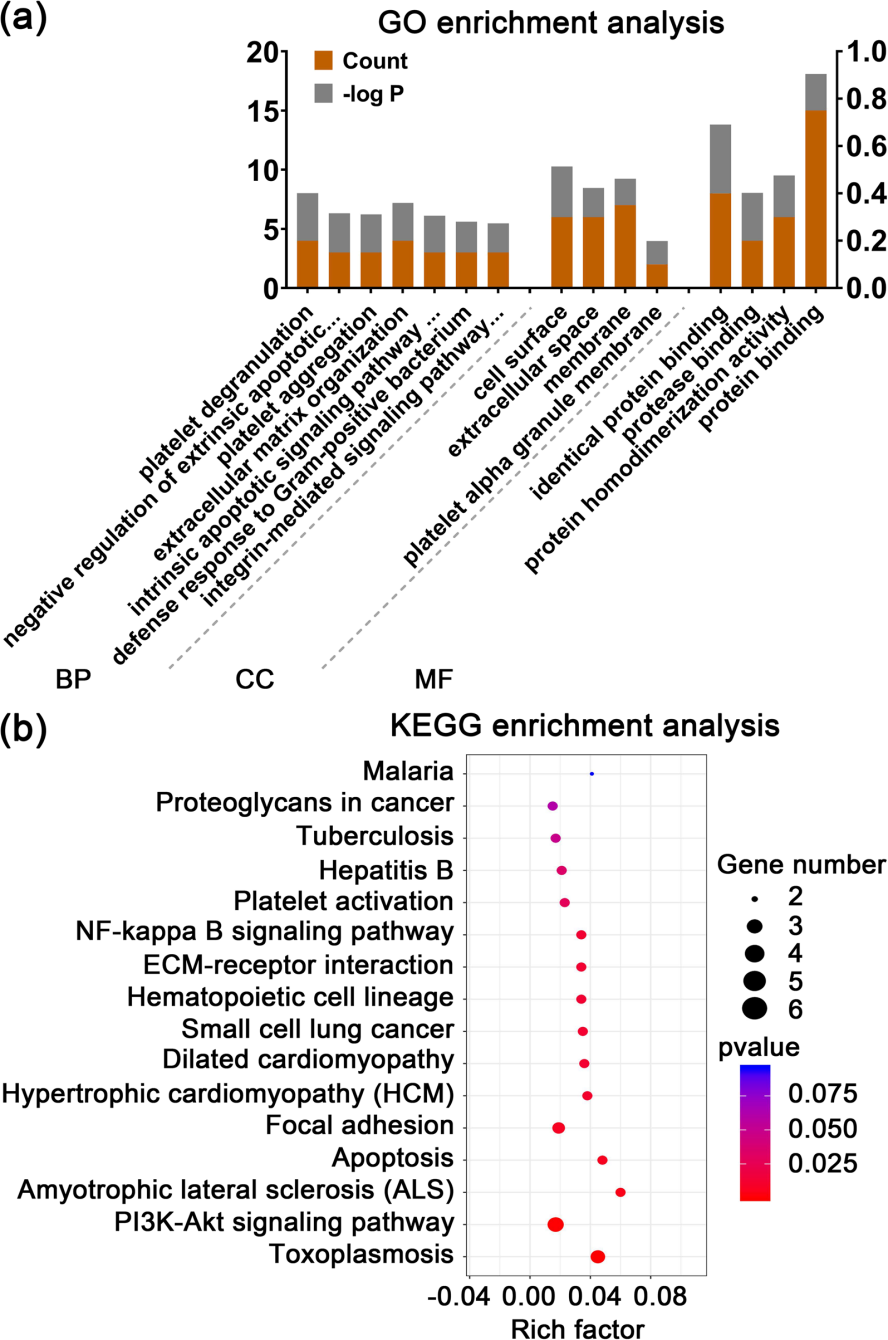
GO and KEGG enrichment analysis of the core targets of DMAG against thrombocytopenia. **a** GO enrichment analysis. GO annotations based on the three-dimension terms of biological process (BP), molecular function (MF), and cellular component (CC). The orange histogram reflects the magnitude of the p value (expressed as − log p). The gray histogram shows the number of genes. **b** KEGG pathway enrichment analysis for the mechanisms of DMAG against thrombocytopenia. A larger richness factor reflects greater enrichment. The size of the bubble represents the number of genes enriched in the different pathways. The colour of the bubble indicates the range of p value

GO and KEGG enrichment analyses were performed to elucidate the underling mechanism of DMAG against thrombocytopenia. GO enrichment analysis showed that the core targets were mainly enriched in platelet degranulation, platelet aggregation, extracellular matrix organization and integrin-mediated signaling pathway (BP), cell surface, extracellular space, membrane and platelet alpha granule membrane (CC), identical protein binding, protease binding, protein homodimerization activity and protein binding (MF) (Fig. 5a), which were important for development and function of megakaryocyte and platelet. KEGG enrichment analysis revealed that the core targets were significantly enriched in PI3K–Akt signaling pathway, hematopoietic cell lineage, ECM-receptor interaction and platelet activation (Fig. 5b), which played a vital role in megakaryocytes differentiation and platelet production.

### Molecular docking stimulation and molecular dynamics simulation verification

**Fig. 6:**
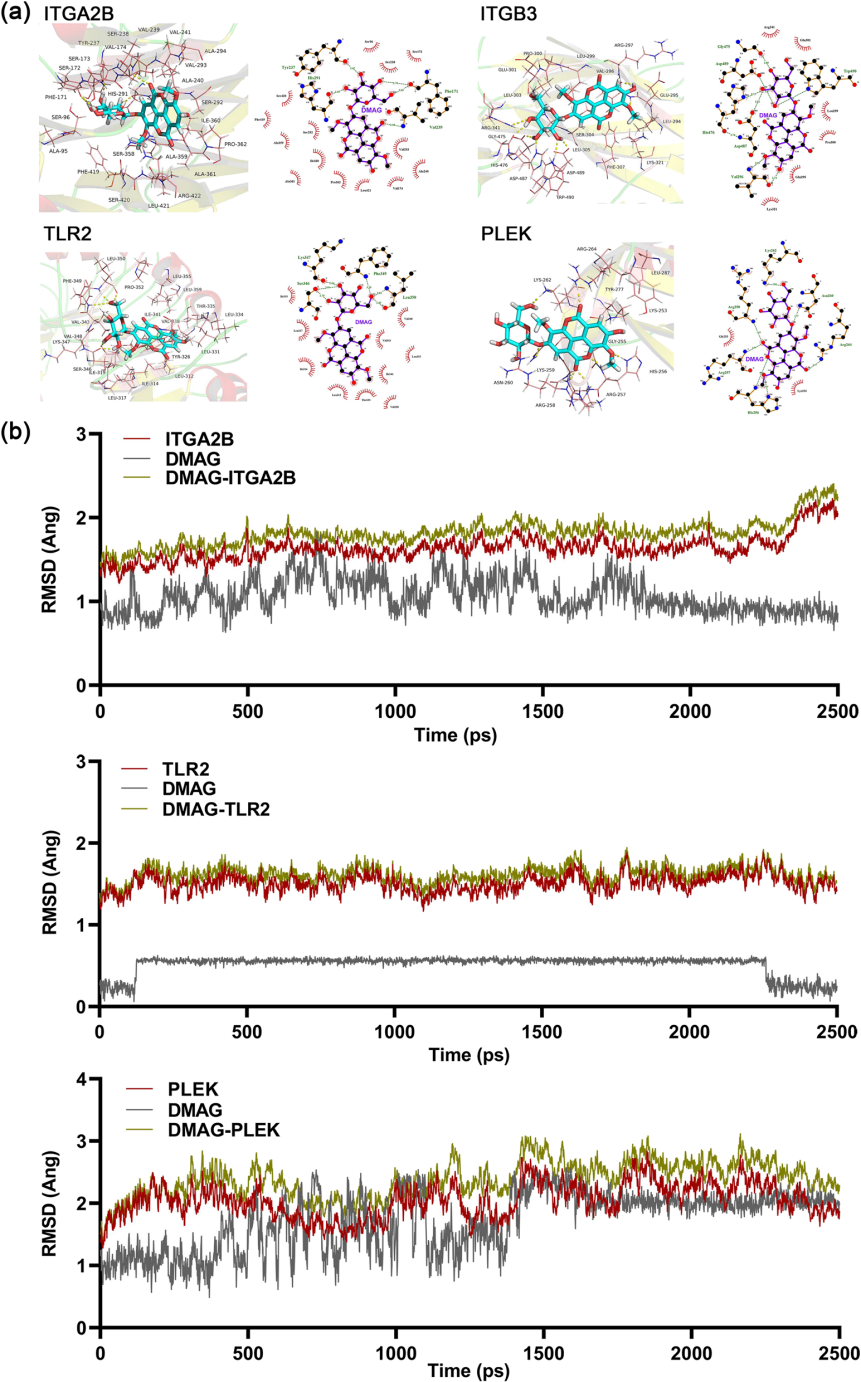
Molecular docking and molecular dynamics simulation show the binding ability between DMAG and its core targets. **a** Detailed interactions of receptors (ITGA2B, ITGB3, TLR2, PLEK) and ligands (DMAG) by molecular docking. The yellow dotted line indicates the interaction between ligand and receptors. **b** The RMSD curves of receptors (ITGA2B, ITGB3, TLR2, PLEK) binding to ligands (DMAG) during 25 ns by molecular dynamics simulation

To further explore the direct relationship between DMAG and its core targets, molecular docking stimulation and molecular dynamics simulation were used to predict their binding possibility. Docking score > 5 kcal mol^−1^ was regarded as a high binding strength. The information of compound-protein docking was listed in Table 1, and visualization for compound-protein combination was shown in Fig. 6a. According to the docking results, the binding scores of ITGB3, ITGA2B, PLEK, TLR2 with DMAG were all greater than 6, indicating that DMAG had a good affinity with the crystal structure of these four core proteins. In molecular dynamics simulation, RSMD represents the root mean square deviation of each atom before and after the binding of receptor and ligand conformations (Badieyan et al. 2012). and we analyzed the stability of the receptor binding to the ligand according to the fluctuation range of RSMD, which showed that the binding of ITGB3, PLEK and TLR2 to DMAG reached equilibrium at about 15 ns, 20 ns, and 20 ns, respectively (Fig. 6b, Additional files 2, 3 and 4). BCL2 and BCL1 have low binding scores with DMAG, but they have performed well in molecular dynamics simulation (Additional file 1: Fig. S2, Additional files 5 and 6). These results suggested that DMAG might directly bind to ITGB3, ITGA2B, TLR2 and PLEK.

**Table 1:** Docking score of DMAG with the core proteins

Proteins	Docking score (kcal mol^−1^)
ITGA2B	6.3757
ITGB3	6.2011
PLEK	6.6829
TLR2	6.8015
BCL2L1	4.7380
BCL2	3.9747

### Validation of the expression of core targets by western blot

**Fig. 7:**
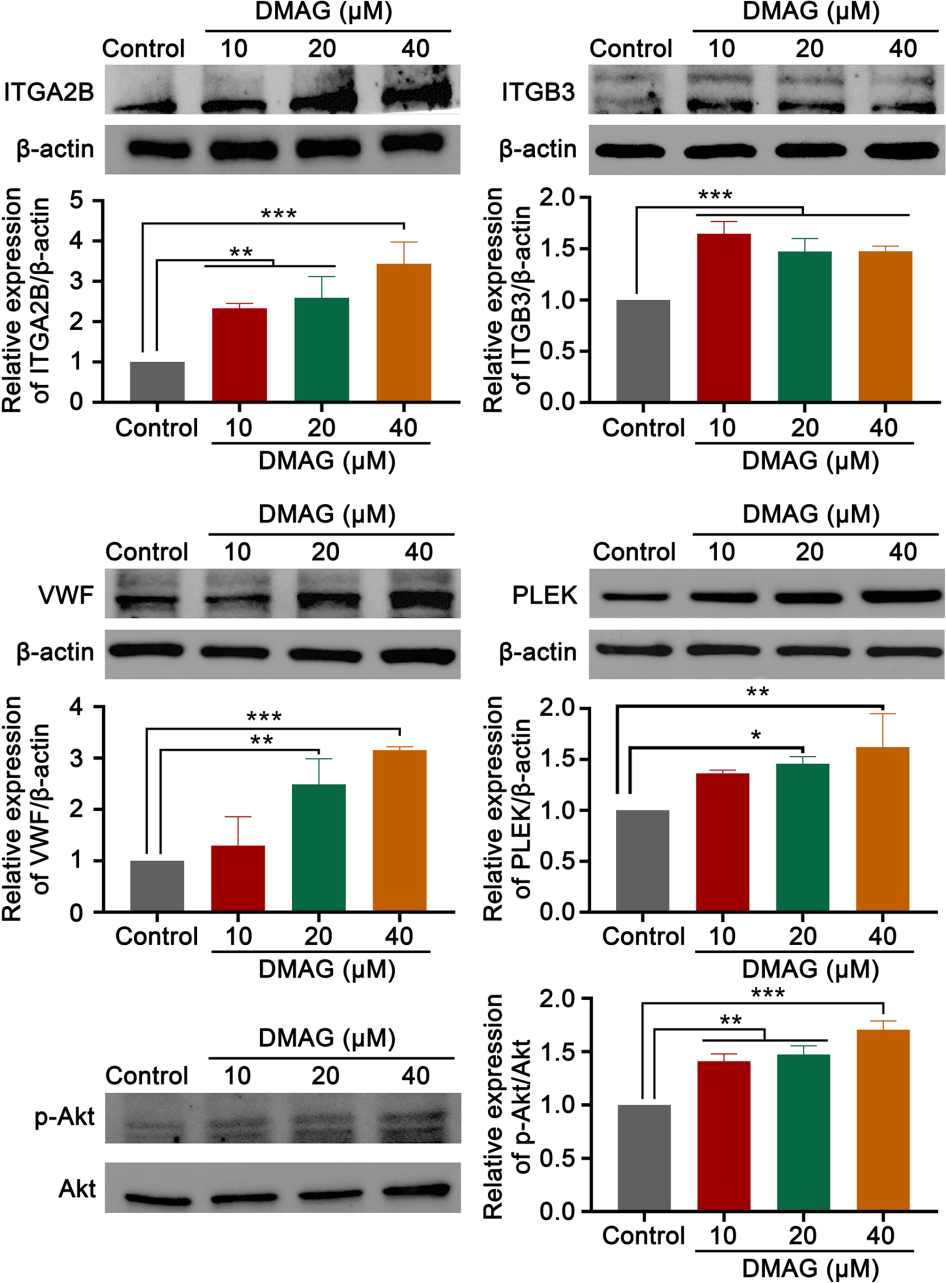
Western blot analysis of expression of core targets of DMAG. The expressions of ITGA2B, ITGB3, VWF, PLEK and p-Akt were detected by western blot after HEL cells were treated with DMAG (10, 20 and 40 μM) for 4 days. The data represent the mean ± SD of three independent experiments; *p < 0.05, **p < 0.01, ***p < 0.001, compared to the control group

According to network pharmacology analysis, the expression of core targets of DMAG against thrombocytopenia was validated by western blot. The results showed that the expressions of ITGA2B, ITGB3, VWF, P-Akt and PLEK, which were related to megakaryocytes differentiation and platelet production were obviously up-regulated induced by DMAG (Fig. 7). The results indicated that DMAG might stimulate megakaryocyte differentiation and platelet production via activation of PI3K–Akt, hematopoietic cell lineage, ECM-receptor interaction and platelet activation signaling pathway.

## Discussion

Thrombocytopenia is a very common blood disorder that is caused by multiple reasons, such as radiotherapy and chemotherapy treatments. Severe thrombocytopenia can lead to bleeding that is fatal. Whoever, there are still no effective and safe drugs for the rapid treatment of thrombocytopenia. SOL, a well known traditional herbal medicine, has long been used for the treatment of various wounds, particularly burns, internal haemorrhage, inflammatory, cancers and metabolic diseases (Zhao et al. 2017). In our previous study, we found that SOL and its ellagic acids had remarkably activities against leukopenia and in promoting megakaryocyte differentiation, respectively (Wang et al. 2020). In the present study, we demonstrated that another ellagic acid derived from SOL, DMAG, significantly promoted megakaryocyte differentiation in vitro and stimulated platelet formation in vivo. Combined with network pharmacology analysis and experimental verification, we explored the targets and elucidated the underling molecular mechanism of DMAG against thrombocytopenia.

We first evaluated the pro-differentiation activity of DMAG in vitro. After the HEL cells were treated with DMAG, the cell size, the expression of megakaryocytes-specific marker CD41, CD61, CD42b and VWF, number of nucleus and DNA ploidy were both increased, indicating DMAG could promote megakaryocyte differentiation. Since megakaryocytes are the precursors of platelets, the acceleration of megakaryocyte differentiation induced by DMAG may be conducive to platelet formation. We thereby identified the therapeutic effects of DMAG on thrombocytopenia. As expected, DMAG administration significantly accelerated platelet recovery in mice with thrombocytopenia induced by X-ray irradiation. The increased number of platelets either caused by enhanced megakaryopoiesis and platelets production, or decreased platelets destruction or clearance. H&E staining and VWF immunohistochemistry results revealed that DMAG elevated the number of BM megakaryocytes in mice with thrombocytopenia. Flow cytometry data demonstrated that DMAG promoted BM megakaryocyte differentiation by increasing the expression of CD41 and CD61. From the H&E staining and VWF immunohistochemistry results, we noticed that the number and size of megakaryocytes in TPO group were bigger than that of the control and DMAG-treated groups. However, the morphology and amounts of megakaryocytes were similar between the control and DMAG-treated groups. The bigger, more mature and greater number of megakaryocytes produced by TPO treatment might result in a much larger number of platelets, which might increase the risk of venous and arterial thrombosis. Although the ability of DMAG in promoting megakaryopoiesis and platelet production was not stronger than that of TPO, the risk of side effects, such as thrombosis of DMAG might be lower when compared with TPO. Furthermore, platelet function was detected to certify whether the platelet induced by DMAG was functional. We found that DMAG administration remarkably recovered damage of platelet function induced by X-ray irradiation. The results of in vitro and in vivo experiments proved that DMAG ameliorated radiation-induced thrombocytopenia in mice at least partly through promotion of megakaryopoiesis, megakaryocyte differentiation and platelet function. This therapeutic effect of DMAG was similar to nanocurcumin, human growth hormone (hGH) and insulin-like growth factor-1 (IGF-1), which promoted megakaryocyte differentiation in vitro and platelet recovery in irradiated mice (Xu et al. 2014; Chen et al. 2018; Mortazavi Farsani et al. 2020).

Network pharmacology was carried out to identify the targets of DMAG against thrombocytopenia. We found eight proteins (ITGA2B, ITGB3, VWF, PLEK, TNF, TLR2, BCL2 and BCL2L1) were the core targets of DMAG for the treatment of thrombocytopenia. Integrins are cell surface receptors that play a crucial role in both platelet activation, adhesion and aggregation (Guidetti et al. 2015). The ITGA2B gene, also called CD41, encodes for the αIIb which exclusive express in megakaryocytes, platelets and some hematopoietic progenitor cells. The ITGB3 gene, also known as CD61, encodes for β3. These two proteins can form a fibrinogen receptor, αIIbβ3, an integrin that is crucial for platelet aggregation through binding of soluble fibrinogen (Nurden et al. 2011). The homozygous mutations in ITGA2B or ITGB3 locus could cause Glanzmann thrombasthenia, a bleeding disorder (Nurden et al. 2013). The heterozygous activating mutations in the membrane-proximal region of the αIIb and β3 subunit could lead to congenital macrothrombocytopenia (Ghevaert et al. 2008; Kunishima et al. 2011; Nurden et al. 2011). Now, CD41 and CD61 were regard as early markers of megakaryocyte differentiation (Psaila et al. 2016). VWF, a large multimeric adhesive glycoprotein, is a well-known mediator of platelet–vessel wall interaction and platelet–platelet interactions under high shear-stress conditions. Reduced or dysfunctional levels of VWF can lead to inherited von Willebrand disease (VWD), an inherited bleeding disorder (Ruggeri 2007). In type 2B VWD, gain-of-function mutations in VWF cause enhanced binding of mutated VWF multimers to platelets through a direct interaction with its receptor GPIba (Bury et al. 2019). Emerging studies had demonstrated that VWF and its receptor played a critical role in megakaryocytopoiesis and platelet production. Abnormalities of GPIb-IX-V expression or an abnormal interaction between newly synthesized VWF with GPIb-IX-V in the megakaryocytes of a family with VWD2B caused by VWF R1308P lead to impaired megakaryocytopoiesis and thrombocytopenia (Nurden et al. 2006, 2010). VWF promoted proplatelet formation (PPF) and platelet production when the human megakaryocytes exposure to high shear rates (Poirault-Chassac et al. 2013). The expression of VWF has been considered as a sensitive and distinct marker for megakaryocytes (Tomer 2004). Pleckstrin (PLEK), a prominent substrate of protein kinase C (PKC) in platelets and leukocytes, has long been considered as a marker of platelet activation (Lian et al. 2009). Previous study had demonstrated that inhibition of PKC by bisindolylmaleimide, GF109203X, a highly selective inhibitor of PKC, suppressed the expression of CD61, phosphorylation of pleckstrin, and megakaryocyte differentiation induced by phorbol 12-myristate 13-acetate (PMA) (Hong et al. 1996). Pleckstrin-null platelets from a pleckstrin null knockout mouse exhibited a marked defect in granule secretion, aggregation, actin polymerization and mild thrombocytopenia (Lian et al. 2009). Toll-like receptors (TLRs) are essential components of the innate immune response and are activated upon interaction with different pathogen-associated molecular patterns (PAMPs). There are 13 TLRs in humans and mice. Among them, TLR1, TLR2, TLR4 and TLR6 are both expressed on megakaryocytes and platelets. TLR2, which forms functional heterodimers with either TLR1 or TLR6, recognizes a wide spectrum of microbial pathogen associated molecules, such as virus, fungal, bacterial pathogens and their ligands (D'Arti and Schattner 2017). It has been reported that stimulation of Meg-01 cells by Pam3CSK4, a specific synthetic TLR2 ligand, activating NF-kappaB, ERK–MAPK, and PI3K/Akt pathways, which leads to up-regulation of transcription factors associated with megakaryocyte maturation, thereby increasing megakaryocyte ploidy. In addition, after the mice are treated with Pam3CSK4, the platelet level initially drops and then returns to normal level, accompanied by an increase in megakaryocyte maturation (Beaulieu et al. 2011). Recent studies have shown that stimulation of Dami cells by heat killed lacto bacillus (HKL), another TLR2 ligand, leading to up-regulation of TLR2 and cytokine production, mainly IL-6, which is essential for megakaryocyte generation and CD41 expression. Additionally, TLR2 induction activates wnt b-catenin signalling pathway components, indicating a cross talk between wnt and TLR pathway leading to megakaryocyte maturation (Undi et al. 2016). BCl-X_L_ is the key pro-survival protein that is essential for survival of megakaryocyte and platelet (Josefsson et al. 2020). Megakaryocyte-specific deletion of BCl-X_L_ in mice triggers megakaryocyte apoptosis and a failure of platelet shedding, which leading to severe thrombocytopenia (Josefsson et al. 2011). The megakaryocytic lineage-specific deletion of both MCL1 and BCL-XL causes embryonic lethality in association with failure of megakaryopoiesis and systemic haemorrhage (Kodama et al. 2012). Tumour Necrosis Factor-a (TNF-a), a pro-inflammatory cytokine, plays an important role in inflammation, anti-tumor responses and homeostasis. It is well known that TNF-a can regulate a wide spectrum of biological processes, such as cell proliferation, differentiation and apoptosis (Tian et al. 2014). These studies demonstrate the critical role of above core targets of DMAG in regulating megakaryocyte differentiation and platelet production. In order to explore the direct relationship between DMAG and its core targets, molecular docking stimulation and molecular dynamics simulation were performed. The results showed that DMAG had a strong binding ability with ITGB3, ITGA2B, PLEK and TLR2, indicating that DMAG might regulate megakaryocyte differentiation and platelet production by directly binding to ITGB3, ITGA2B, PLEK and TLR2.

Through GO enrichment analysis, we found that the above targets were mainly associated with platelet degranulation, platelet aggregation, extracellular matrix organization and integrin-mediated signaling pathway (BP), cell surface, extracellular space, membrane and platelet alpha granule membrane (CC), identical protein binding, protease binding, protein homodimerization activity and protein binding (MF), which were all involved in development and function of megakaryocyte and platelet (Bianchi et al. 2016; Eto and Kunishima 2016; Leiva et al. 2018). According to KEGG enrichment analysis results, the core targets of were mainly enriched in PI3K–Akt signaling pathway, hematopoietic cell lineage, ECM-receptor interaction and platelet activation. PI3K–Akt pathway is one of the most crucial signaling pathways that is activated in response to various types of stimulations, including cytokines, growth factors, hormones, integrin and ECM proteins. This pathway regulates a wide spectrum of cellular processes, such as cell cycle, proliferation, survival, differentiation and death (Guidetti et al. 2015; Moroi and Watson 2015). It is known that PI3K–Akt pathway is located downstream of TPO/MPL signaling pathway, activating several transcription factors to derive megakaryocyte differentiation and maturation, as well as platelet production (Bianchi et al. 2016). Emerging evidences have demonstrated that PI3K–Akt pathway is also activated by several platelet receptors, including GPIb-IX-V, ITAM-bearing receptors, G-protein-coupled receptors, and integrins, that regulates platelet activation and haemopoiesis (Guidetti et al. 2015). The ECM is the non-cellular structure that provide tissue cohesion and rigidity. The BM ECM is crucial for normal hematopoiesis. It contains variety of proteins, including fibrinogen, collagens, fibronectin and laminin, as well as multiple soluble proteins, such as chemokines, cytokines and secreted enzymes. Different ECM components possess diverse function in regulating megakaryocyte development and platelet formation (Leiva et al. 2018). For instance, type III and IV collagens stimulate megakaryocyte maturation and platelet production via the PI3K/Akt signaling pathway (Abbonante et al. 2017). Fibrinogen binds to αIIbβ3 on the surface of megakaryocyte to stimulate proplatelet formation and platelet release (Larson and Watson 2006). In the present study, through western blot validation of the expression of core targets, we found that these signaling pathways were activated by DMAG. More importantly, we also detected the expression of Akt, a core protein in PI3K/Akt signaling pathway. We found that the phosphorylation of Akt were activated by DMAG, indicating that PI3K/Akt signaling pathway might be the main signaling pathway mediated DMAG induced megakaryocyte differentiation.

Based on previous studies and our findings, we can conclude that DMAG promotes megakaryocyte differentiation and platelet production via directly binding to ITGB3, ITGA2B and TLR2, thereby activation of PI3K–Akt signaling pathway, hematopoietic cell lineage, ECM-receptor interaction and platelet activation.

## Conclusions

In summary, the present study demonstrated that DMAG, a natural ellagic acid derived from SOL could significantly promote megakaryocyte differentiation in vitro and enhance platelet count and function in mice with thrombocytopenia. Furthermore, via network pharmacology method and experimental verification, our study also confirmed that the anti-thrombocytopenia activity of DMAG might be mediated by activating PI3K–Akt signaling pathway, hematopoietic cell lineage, ECM-receptor interaction and platelet activation. Altogether, our results suggest that DMAG may be a curative agent for the treatment of thrombocytopenia.

## Supplementary Information

**Additional file 1: Figure S1.** Identification of DMAG from SOL. (a) Total ion chromatogram of SOL; (b) UV chromatogram at 254 nm of DMAG; (c) Fragmentation patterns of DMAG. **Figure S2.** Molecular docking and molecular dynamics simulation show the interaction between DMAG and its core targets. (a) Detailed interactions of receptors (BCL2 and BCL2L1) and ligands (DMAG) by molecular docking. The yellow dotted line indicates the interaction between ligand and receptors. (b) The RMSD curves of receptors (BCL2 and BCL2L1) binding to ligands (DMAG) during 25 ns by molecular dynamics simulation. **Figure S3.** Origin data of Western blot analysis in Fig. 7.

**Additional file 2: GIF S1.** The GIF of molecular dynamics simulation of DMAG with ITGA2B.

**Additional file 3: GIF S2.** The GIF of molecular dynamics simulation of DMAG with PLEK.

**Additional file 4: GIF S3.** The GIF of molecular dynamics simulation of DMAG with TLR2.

**Additional file 5: GIF S4.** The GIF of molecular dynamics simulation of DMAG with BCL2.

**Additional file 6: GIF S5.** The GIF of molecular dynamics simulation of DMAG with BCL2L1.

## Data Availability

The raw data supporting the conclusions of this manuscript will be made available by the authors, without undue reservation, to any qualified researcher.

## Abbreviations

DMAG: 3,3ʹ-Di-*O*-methylellagic acid 4ʹ-glucoside
SOL: *Sanguisorba officinalis* L.
GO: Gene Ontology
KEGG: Kyoto Encyclopedia of Genes and Genomes
MK: Megakaryocytes
HSCs: Hematopoietic stem cells
TPO: Thrombopoietin
BM: Bone marrow
ECM: Extracellular matrix
ATRs: Allergic transfusion reactions
TPO-RA: Thrombopoietin receptor agonists
HCS: High content screening
PRP: Platelet-rich plasma
PPP: Platelet-poor plasma
PPI: Protein–protein interaction
BC: Betweenness centrality
CC: Closeness centrality
MPV: Mean platelet volume
PLT: Platelet
VWF: Von Willebrand factor
VWD: Von Willebrand disease
PLEK: Pleckstrin
PKC: Protein kinase C
TLRs: Toll-like receptors
