# Supplementary material for: DMAG, a novel countermeasure for the treatment of thrombocytopenia
Source: Mol Med. 2021 Nov 27;27:149. doi: 10.1186/s10020-021-00404-1 (PMC8626956; doi:10.1186/s10020-021-00404-1)
Supplement: Supplementary file 1 — Additional file 1: Figure S1. Identification of DMAG from SOL. (a) Total ion chromatogram of SOL; (b) UV chromatogram at 254 nm of DMAG; (c) Fragmentation patterns of DMAG. Figure S2. Molecular docking and molecular dynamics simulation show the interaction between DMAG and its core targets. (a) Detailed interactions of receptors (BCL2 and BCL2L1) and ligands (DMAG) by molecular docking. The yellow dotted line indicates the interaction between ligand and receptors. (b) The RMSD curves of receptors (BCL2 and BCL2L1) binding to ligands (DMAG) during 25 ns by molecular dynamics simulation. Figure S3. Origin data of Western blot analysis in Fig. 7. [file 10020_2021_404_MOESM1_ESM.docx]

Additional files


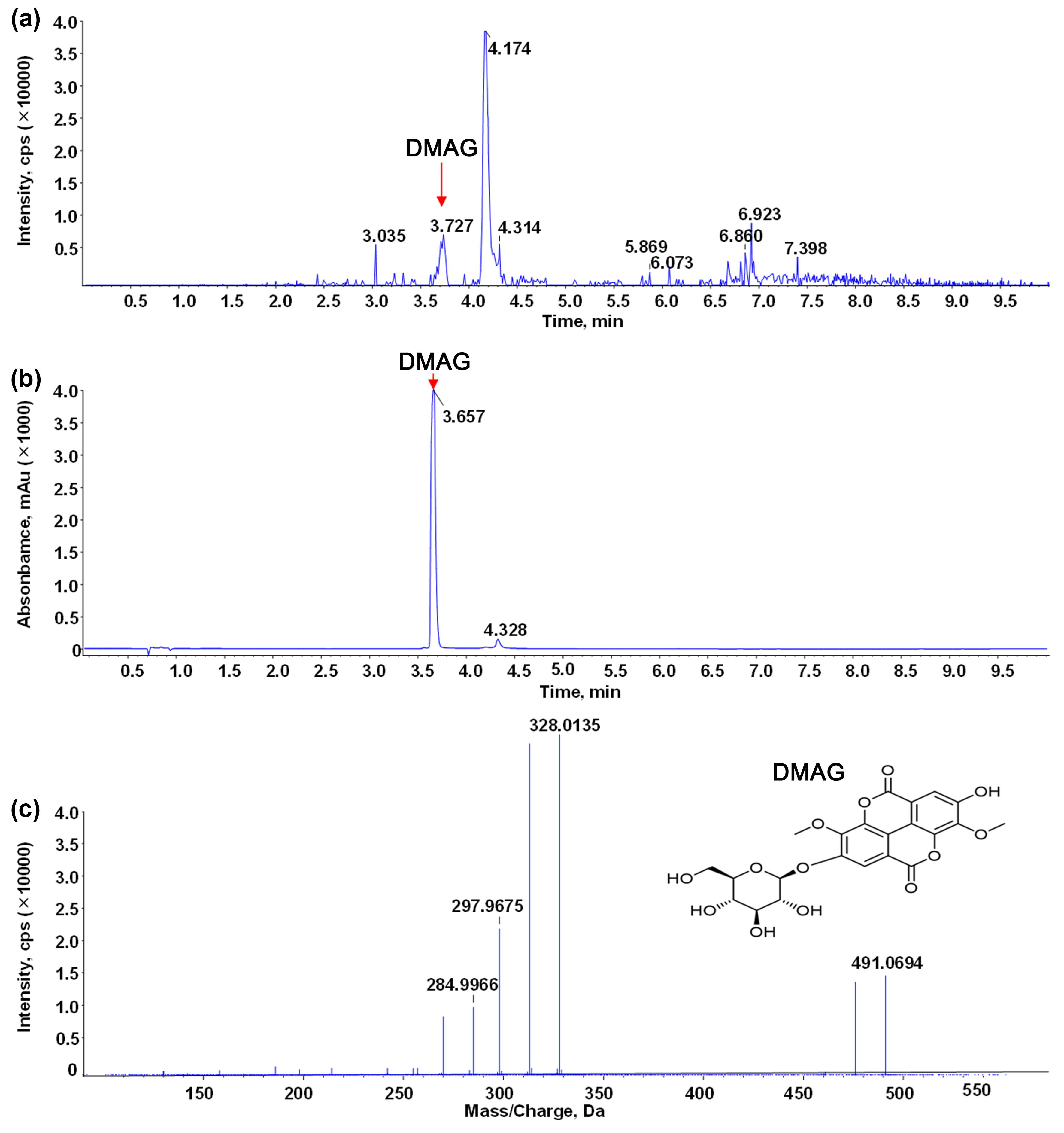


**Figure S1.** Identification of DMAG from SOL. (a) Total ion chromatogram of SOL; (b) UV chromatogram at 254nm of DMAG; (c) Fragmentation patterns of DMAG.


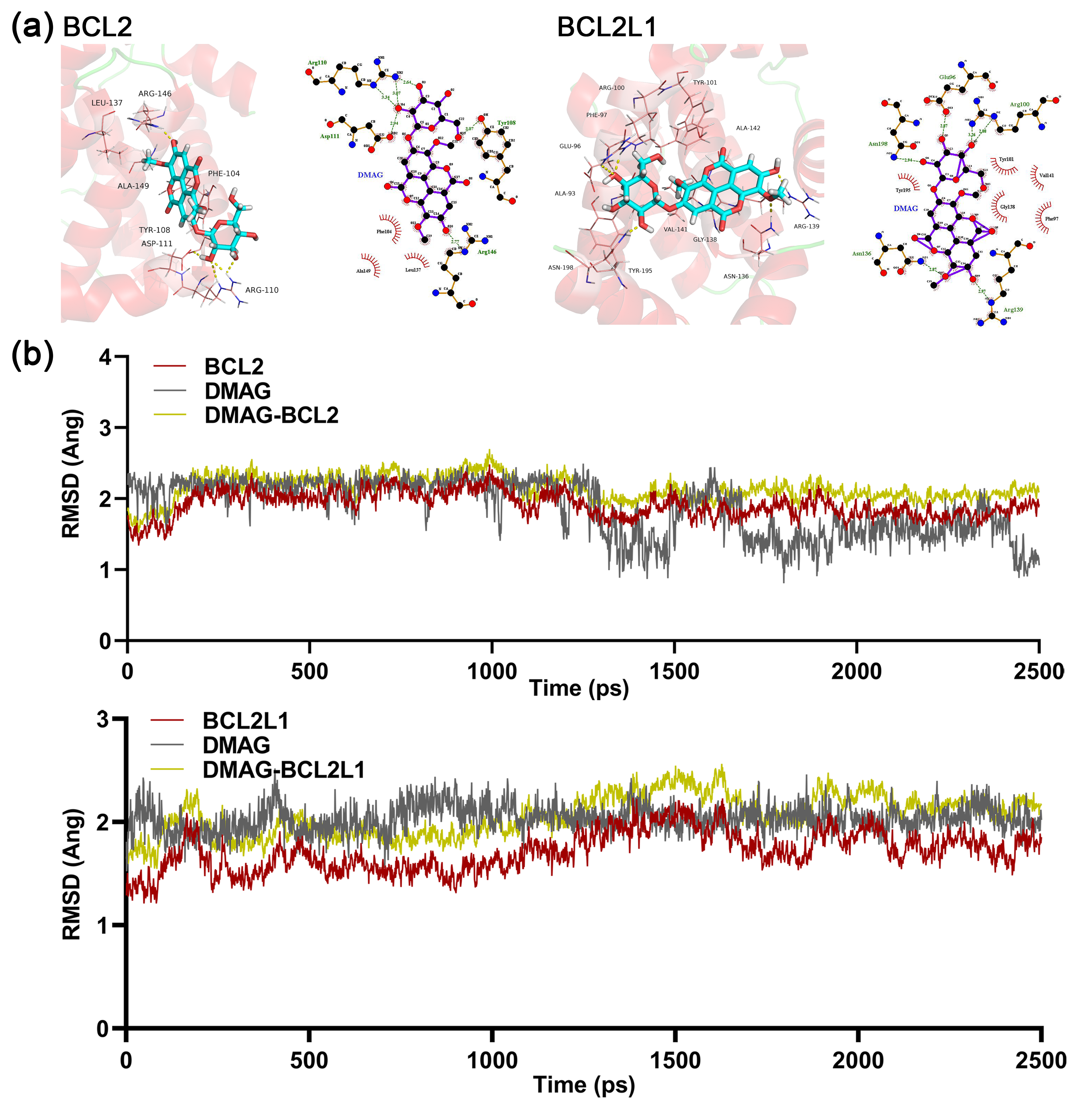


**Figure S2.** Molecular docking and molecular dynamics simulation show the interaction between DMAG and its core targets. (a) Detailed interactions of receptors (BCL2 and BCL2L1) and ligands (DMAG) by molecular docking. The yellow dotted line indicates the interaction between ligand and receptors. (b) The RMSD curves of receptors (BCL2 and BCL2L1) binding to ligands (DMAG) during 25 ns by molecular dynamics simulation.


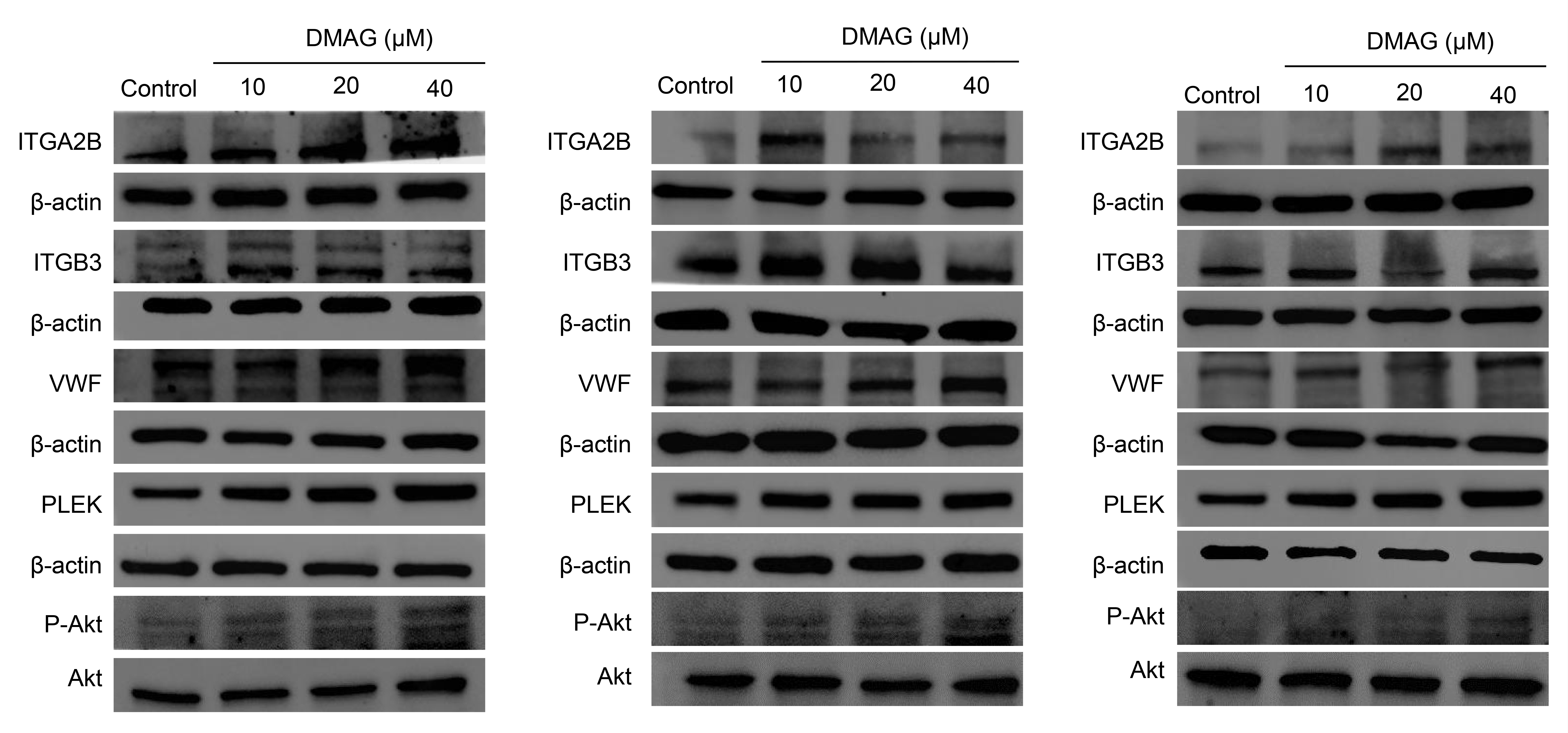


**Figure S3.** Origin data of Western blot analysis in Figure 7.
