# Supplementary figures and images for: DMAG, a novel countermeasure for the treatment of thrombocytopenia
Source: Mol Med. 2021 Nov 27;27:149. doi: 10.1186/s10020-021-00404-1 (PMC8626956; doi:10.1186/s10020-021-00404-1)

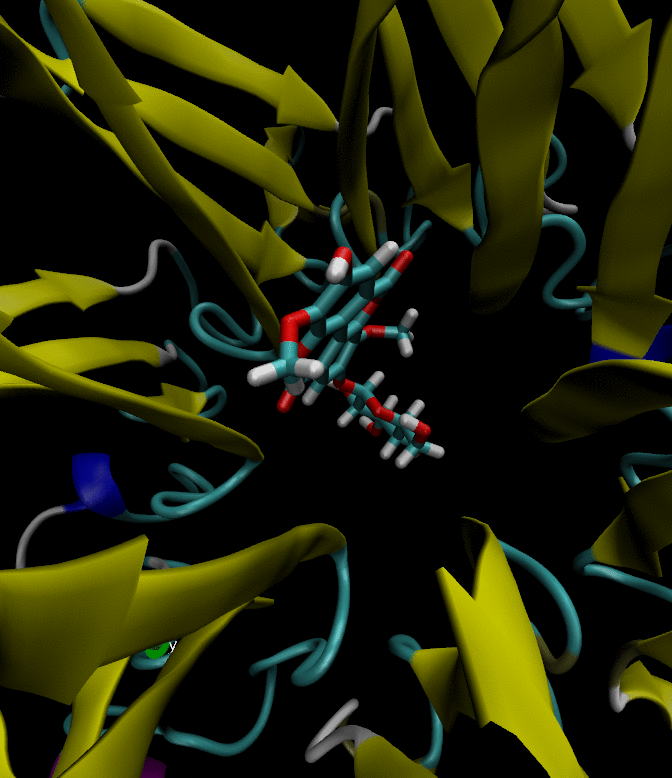

Supplement: Supplementary file 2 — Additional file 2: GIF S1. The GIF of molecular dynamics simulation of DMAG with ITGA2B. [file 10020_2021_404_MOESM2_ESM.gif]

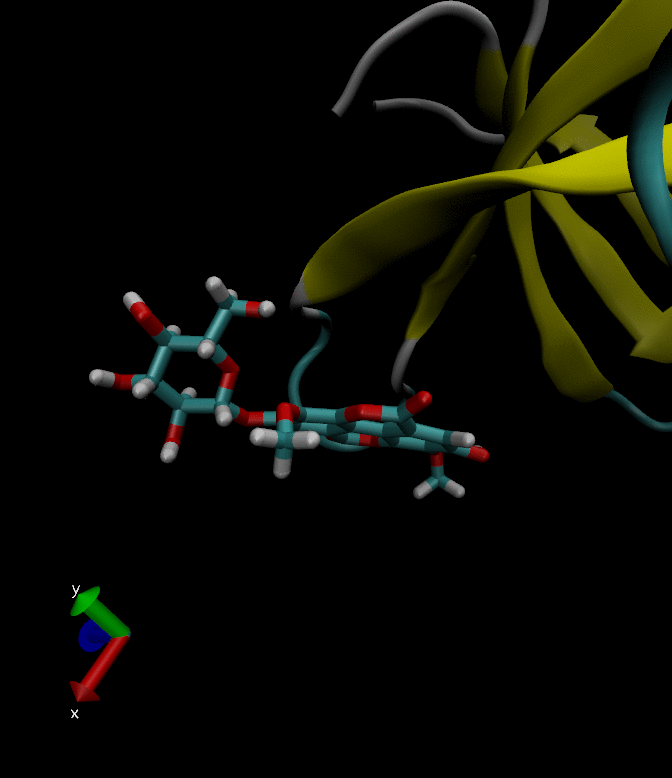

Supplement: Supplementary file 3 — Additional file 3: GIF S2. The GIF of molecular dynamics simulation of DMAG with PLEK. [file 10020_2021_404_MOESM3_ESM.gif]

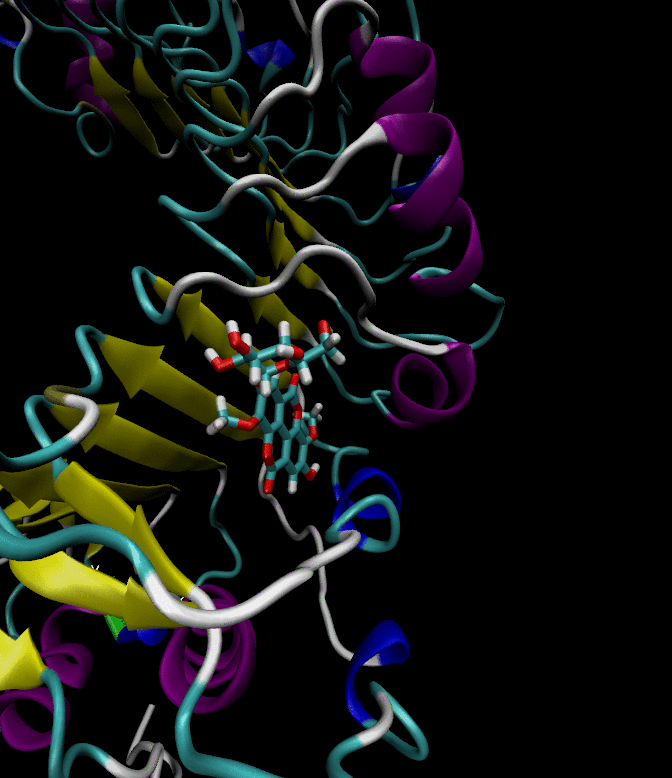

Supplement: Supplementary file 4 — Additional file 4: GIF S3. The GIF of molecular dynamics simulation of DMAG with TLR2. [file 10020_2021_404_MOESM4_ESM.gif]

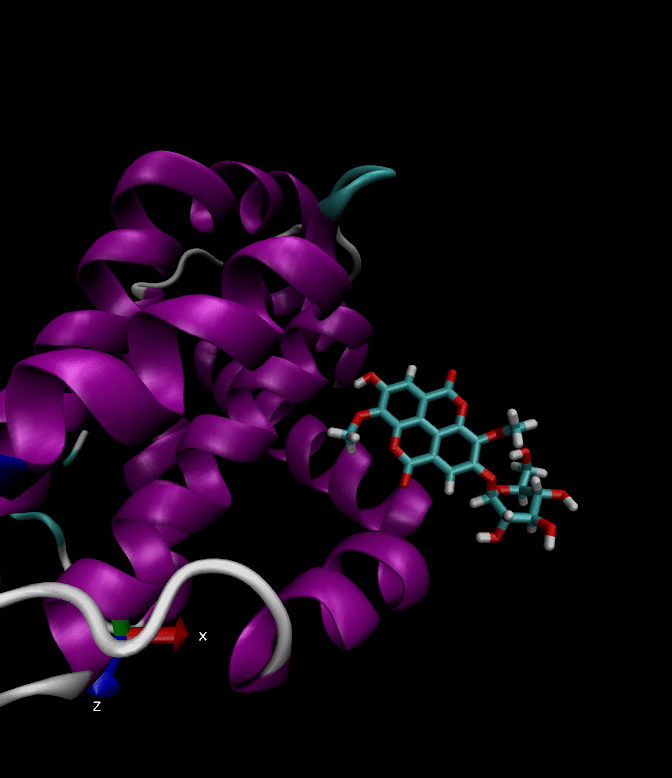

Supplement: Supplementary file 5 — Additional file 5: GIF S4. The GIF of molecular dynamics simulation of DMAG with BCL2. [file 10020_2021_404_MOESM5_ESM.gif]

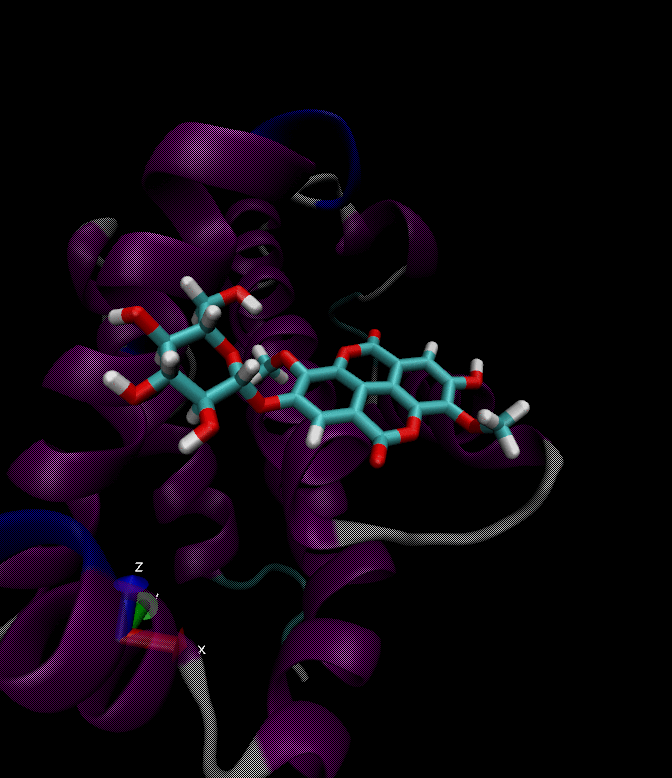

Supplement: Supplementary file 6 — Additional file 6: GIF S5. The GIF of molecular dynamics simulation of DMAG with BCL2L1. [file 10020_2021_404_MOESM6_ESM.gif]
